# Neonatal Sepsis in a Resource-Limited Setting: Causative Microorganisms and Antimicrobial Susceptibility Profile

**DOI:** 10.1155/2022/7905727

**Published:** 2022-05-27

**Authors:** Edna Nai Acheampong, Joseph Adjei Tsiase, Daniel Kwame Afriyie, Seth Kwabena Amponsah

**Affiliations:** ^1^Greater Accra Regional Hospital, Accra, Ghana; ^2^Department of Pharmacy, Ghana Police Hospital, Accra, Ghana; ^3^Department of Medical Pharmacology, University of Ghana Medical School, Accra, Ghana

## Abstract

**Background:**

Empiric treatment of suspected neonatal sepsis must be based on data on setting-specific causative pathogens and their respective susceptibilities to antimicrobials, as well as universal treatment guidelines. This approach will ensure better therapeutic outcomes and reduce mortality.

**Objectives:**

The objectives of this study were to determine the bacteriological profile and antibiotic susceptibility pattern of isolated microorganisms responsible for neonatal sepsis in a regional hospital in Ghana.

**Methods:**

This was a retrospective study that assessed causative microorganisms and antimicrobial susceptibility profiles of neonates suspected of sepsis at the Greater Accra Regional Hospital from January 2018 to December 2019. Blood culture was done using a fully automated BACTEC 9240 blood culture system. Bacteria isolates were identified by Gram staining and conventional biochemical methods. Antimicrobial susceptibility testing was done by Kirby–Bauer's disc diffusion method, and interpretations were carried out according to clinical and laboratory standards. Culture and antibiotic sensitivity reports were obtained and the data subsequently analyzed.

**Results:**

Of 2514 blood samples collected from neonates suspected of neonatal sepsis, 528 (21.0%) of the samples were found to be culture-positive. The majority of these positive cultures were from male neonates (68.9%). A total of 11 different pathogens were isolated, of which Gram-positive organisms had a preponderance of 72.0% over Gram-negative organisms (28.0%). *Staphylococcus epidermidis* was the most common pathogen identified, accounting for 60.0% of isolates. The most prevalent Gram-negative bacteria were *Klebsiella spp.* (13.6%). Most Gram-positive microorganisms showed sensitivity to amikacin, meropenem, vancomycin, and piperacillin/tazobactam. Gram-positive isolates were found to be resistant to ampicillin and penicillin, but moderately susceptible to flucloxacillin. Most Gram-negative isolates were sensitive to meropenem.

**Conclusion:**

The prevalence of culture-proven sepsis was 21.0%. The most prevalent Gram-negative bacteria were *Klebsiella spp*. As there is some level of antibiotic resistance observed in the current study, it is necessary for routine microbial analysis of samples and their antibiogram.

## 1. Introduction

Neonatal sepsis is one of the most important causes of mortality in developing countries and yet the most preventable [1]. Neonatal sepsis is known to contribute significantly to under-five deaths [2]. Survival of neonatal sepsis may be associated with serious long-term morbidities such as cerebral palsy, psychomotor delay, auditory impairment, and bronchopulmonary dysplasia [3].

In developing countries, the incidence of bloodstream infection is estimated to be around 5.5 per 1000 live births [4]. Data on the prevalence of neonatal sepsis in sub-Saharan Africa are limited by uncertainty with diagnosis. Generally, neonatal infections in resource-poor settings can be high because births occur at home, often without skilled attendants to facilitate delivery [5]. Furthermore, diagnosis of sepsis in neonates is complicated by many factors, including nonspecific clinical symptomatology [6]. This often leads to a delay in the decision to seek medical care and/or treatment. The standard of care associated with neonatal sepsis includes empiric antibiotic treatment [7]. Usually, the choice of an antibiotic for infection treatment (empiric) must be based on commonly isolated causative microorganisms and antibiotic susceptibility patterns [8].

Indeed, there are geographical and temporal differences in the etiology of neonatal infections [9]. Recent reviews have shown that in developing countries, Gram-negative microorganisms predominate in early-onset sepsis (EOS) among infants, with *Escherichia coli* being the most commonly isolated pathogen [7]. In a study conducted in Ghana, common bacteria isolated from neonates with infection included coagulase-negative staphylococcus (CoNS), *Staphylococcus aureus*, and *Streptococcus species* [10]. In another related study in Ghana, *S. epidermidis*, *S. aureu*s, *Pseudomonas aeruginosa*, *E. coli*, and *Proteus mirabilis* were isolated [11].

The fear of missing diagnosis of neonatal sepsis is high. Therefore, prompt empirical antibiotic therapy based on risk-factor-driven decisions and clinical symptoms has been shown to reduce mortality in neonates [12]. The World Health Organization (WHO) has provided guidelines for the management of infection among infants in resource-poor settings [13]. These guidelines recommend hospitalization and administration of a combination of aminoglycoside and benzylpenicillin or ampicillin in infants (less than 2 months) with clinical symptoms indicative of infection for at least 7–10 days [13]. Although there have been previous studies on causative microorganisms of neonatal sepsis and their antibiotic susceptibility, there is a paucity of data at the Greater Accra Regional Hospital. Therefore, this study sought to obtain data on the bacteriological profile and antimicrobial susceptibility pattern of culture isolates from neonates with sepsis at the Greater Accra Regional Hospital, Ghana.

## 2. Materials and Methods

### 2.1. Study Design

This was a retrospective study that analyzed data of blood culture and antibiotic sensitivity reports of neonates with suspected sepsis at the Greater Accra Regional Hospital, Ghana, from January 2018 to December 2019. Culture and antibiotic sensitivity reports were obtained from the Bacteriology Unit of the hospital.

### 2.2. Study Site

The study was conducted at the Greater Accra Regional Hospital (Accra, Ghana), one of the hospitals under the Ghana Health Service. The Greater Accra Regional Hospital, previously called Ridge Hospital, was established in 1928 by the British Colonial Government. The hospital received regional hospital status in 1997.

This hospital is located in Accra, the capital city of Ghana, under the Accra Metropolis of the Ghana Health Service. The Greater Accra Regional Hospital can be located on latitude 5.562679 and longitude -0.1989727. It is within the Osu Klottey submetropolitan.

The Greater Accra Regional Hospital has 45 specialist doctors, 39 medical doctors (of various grades ranging from Deputy Chief Medical Officer to Medical Officer), 65 senior house officers, 35 house officers, 17 pharmacists, and 467 nurses, among others. The key functions of this 191-bed hospital (which is being refurbished into a 623-bed capacity) include specialist support services for all healthcare institutions in the Greater Accra Region, the provision of accident and emergency services, pharmaceutical services, laboratory services, and the provision of medical supplies and consumables to patients.

The hospital provides the following outpatient services: eye, dental, ear, nose, and throat clinics, diabetic/hypertensive clinic, and general outpatient clinic, among others. The inpatient units of the hospital include Neonatal Intensive Care, Surgical, Children, and Medical. The laboratory department of the hospital has 6 subunits, namely, clinical chemical pathology, hematology, blood bank, parasitology, bacteriology, virology, and immunology.

### 2.3. Study Population

Neonates suspected of sepsis and admitted to the Neonatal Intensive Care Unit of the Department of Child Health, Greater Accra Regional Hospital, were the study population. Blood samples were collected from these neonates for culture and antibiotic susceptibility testing. For the purpose of this study, neonatal sepsis is defined as neonates (up to 28 days) presenting with one or more of the following features: temperature instability (presence of fever (≥38°C) or hypothermia (≤36°C)), hemodynamic instability, convulsion, lethargy, feeding intolerance, hypoglycemia, vomiting, bulging fontanels, respiratory distress, jaundice, and signs of infection on the skin and/or umbilical pus discharge. EOS is defined as sepsis occurring within the first 48–72 hours of life, and late-onset sepsis (LOS) occurs after 72 hours of life of a neonate (usually up to 28 days).

### 2.4. Bacteria Identification and Antibiotic Susceptibility Tests

Blood culture was done using a fully automated BACTEC 9240 blood culture system (Becton Dickinson Diagnostic Instrument Systems, Sparks, Maryland). Isolates from positive bottles were subcultivated and identified using biochemical methods [14]. In brief, Gram-positive bacteria were identified by catalase, slide, and tube coagulase tests and Gram-negative bacteria by API 20E and 20NE (BioMerieux, France). Antibiotic susceptibility tests were done using Kirby–Bauer's disc diffusion method on Mueller–Hinton agar (Oxoid, UK), in accordance with Clinical Laboratory Standards Institute (CLSI) criteria.

### 2.5. Data Collection

Data on blood culture and antibiotic susceptibility were obtained from the Bacteriology Unit of the Laboratory Department, Greater Accra Regional Hospital. This information was subsequently evaluated for clinical relevance by 2 senior medical doctors, 2 pharmacists, and 1 microbiologist. Data on blood culture isolates and their susceptibility patterns were collected, tabulated, and analyzed. Demographic data on all neonates were also captured.

### 2.6. Data Analysis

Data were presented as descriptive statistics (frequency tables, percentages, and charts). Data analysis was carried out using Microsoft Excel 2010.

### 2.7. Ethical Consideration

Approval for the conduct of this research was obtained from the Greater Accra Regional Hospital Administration.

## 3. Results

### 3.1. Prevalence of Neonatal Sepsis

A total of 2514 neonates with suspected sepsis had their blood samples collected. Blood culture reports showed that 528 samples (21.0%) were positive. Of the 528 culture-proven positive cases, 364 (68.9%) were obtained from males, whilst 164 (31.1%) were from females. Of 528 clinical and laboratory-confirmed cases, 357 (67.6%) had EOS and 171 (32.4%) had LOS. Details of bacteria isolated from blood cultures are presented in Table 1.

### 3.2. Bacteria Isolates

**Figure 1:**
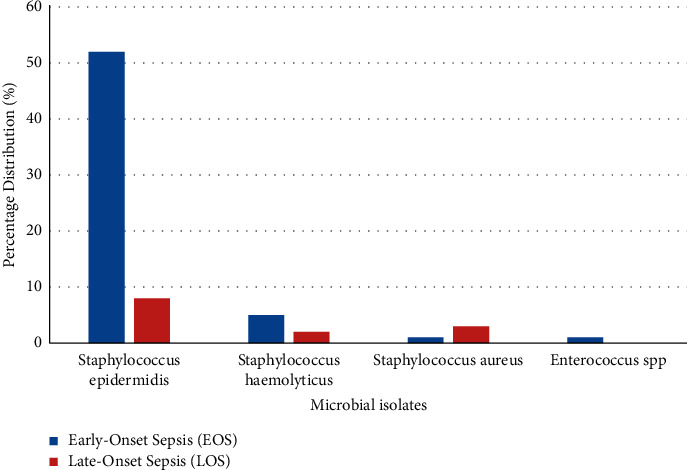
Distribution of Gram-positive bacteria in EOS and LOS.

**Figure 2:**
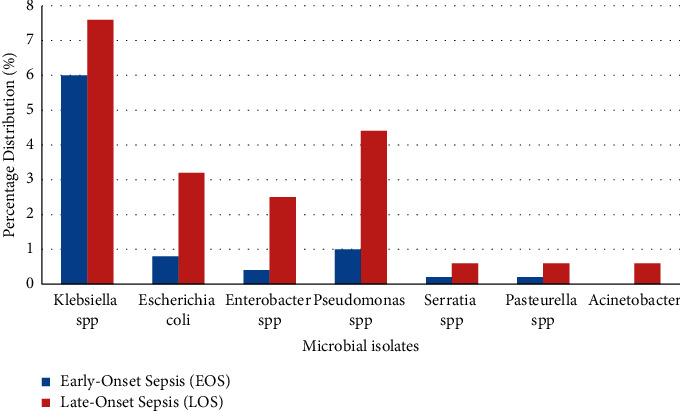
Distribution of Gram-negative bacteria in EOS and LOS.

The 528 isolates consisted of 11 microorganisms. A total of 380 (72.0%) were Gram-positive, whilst 148 (28.0%) were Gram-negative. Gram-positive and Gram-negative bacteria isolates per EOS and LOS distribution are presented in Figures 1 and 2.

*Staphylococcus epidermidis, Klebsiella spp., Staphylococcus haemolyticus, Pseudomonas aeruginosa, Staphylococcus aureus, Escherichia coli,* and *Enterobacter spp*. constituted the top 7 isolates. *S. epidermidis* was the highest causative organism in EOS, whilst *Klebsiella spp.* was the major cause of LOS. Overall, *S. epidermidis* constituted 60.0% of the isolates and *Klebsiella spp.* constituted 13.6%.

### 3.3. Antimicrobial Susceptibility Pattern among Isolated Gram-Positive Microorganisms

**Figure 3:**
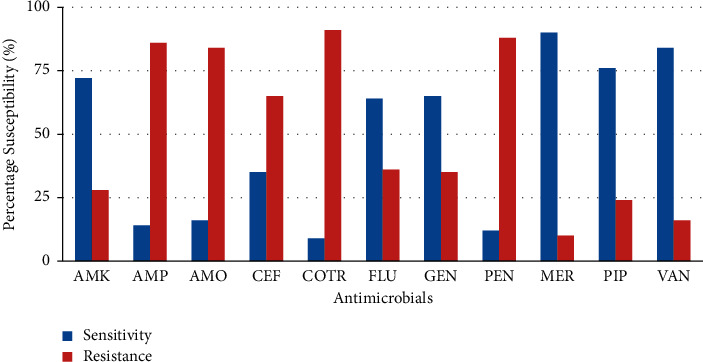
Overall antimicrobial sensitivity/resistance pattern of Gram-positive isolates. Key: AMK, amikacin; AMP, ampicillin; AMO, amoxiclav; CEF, cefuroxime; CO-TR, cotrimoxazole; FLU, flucloxacillin; GEN, gentamicin; PEN, penicillin; MER, meropenem; P/T, piperacillin/tazobactam; VAN, vancomycin.

Based on antibiotic susceptibility testing of isolated Gram-positive microorganisms, most showed sensitivity to amikacin, meropenem, vancomycin, and piperacillin/tazobactam. Gram-positive isolates demonstrated fairly good sensitivity to flucloxacillin (64.0%) and gentamicin (65.0%). *S. epidermidis* and *S. haemolyticus* were resistant to ampicillin (85.9%) and penicillin (88.1%), which are commonly used antibiotics in neonatal sepsis treatment. In addition, *S. aureus* showed resistance (89.9%) to ampicillin and penicillin (86.0%). Gram-positive isolates were also found to be resistant to cotrimoxazole, amoxiclav, and cefuroxime. The overall antimicrobial susceptibility pattern among isolated Gram-positive bacteria is presented in Figure 3.

### 3.4. Antimicrobial Susceptibility Pattern among Isolated Gram-Negative Microorganisms

**Figure 4:**
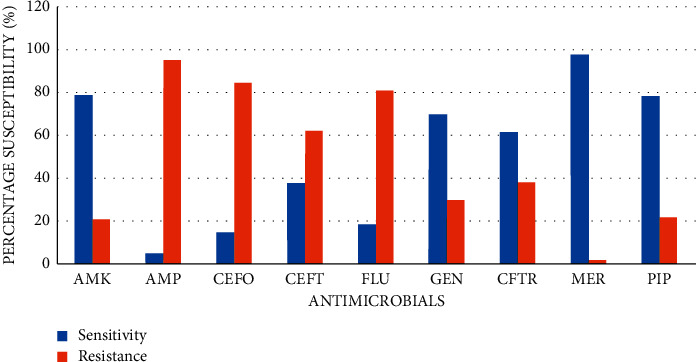
Overall antimicrobial sensitivity/resistance pattern of Gram-negative isolates. Key: AMK, amikacin; AMP, ampicillin; AMO, amoxiclav; CEF, cefuroxime; CO-TR, cotrimoxazole; FLU, flucloxacillin; GEN, gentamicin; PEN, penicillin; MER, meropenem; P/T, piperacillin/tazobactam.

*Klebsiella spp.* demonstrated susceptibility to the beta-lactam antibiotics, meropenem (97.0%) and piperacillin-tazobactam (76.8%). However, *Klebsiella spp.* showed relatively high resistance to ampicillin (89.2%) and cefotaxime (82.0%). Among non-beta-lactam antibiotics, *Klebsiella spp.* showed high sensitivity to amikacin (95.0%). *P. aeruginosa* showed relatively high resistance to ampicillin (89.1%) and cefotaxime (71.0%). *E. coli* demonstrated moderate sensitivity to commonly used antibiotics: gentamicin (62.0%), amikacin (71.0%), and ceftriaxone (67.3%). *E. coli* showed relatively high sensitivity to meropenem (94.7%) and piperacillin/tazobactam (62.0%). *Enterobacter* species demonstrated high sensitivity to meropenem (92.8%) but also showed high resistance to ampicillin (87.3%), cefotaxime (80.0%), flucloxacillin (67.1%), and ceftazidime (60.1%). A summary of the overall antimicrobial susceptibility pattern among isolated Gram-negative microorganisms is shown in Figure 4.

## 4. Discussion

In this study, 528 of 2514 blood samples taken from neonates suspected of sepsis showed culture positivity and a prevalence rate of 21.0%. Bacteria are the most common etiological agents implicated in neonatal sepsis; however, other organisms other than bacteria like adenovirus, enterovirus, coxsackievirus, rubella virus, *Toxoplasma* species, and *Candida* species have been implicated [15]. A study revealed that negative blood cultures are not an indication of the absence of infection and that about 26.0% of all neonatal sepsis cases could be attributed to anaerobes [16]. In addition, a low prevalence of sepsis based on blood culture positivity could be attributed to limitations of laboratory setup (equipment, reagents, or skills) to identify very low bacteremia, fungi, and viruses [17].

Results from this study showed that most neonates had EOS (67.6%), compared to late-onset septicemia (32.4%). A similar observation was made in a related study done by Priyadarshini et al. [18], in which EOS was found to be 64.0%. Other studies have found similar trends with greater proportions of EOS than LOS [19–21]. This could be attributed to prematurity, low birth weight, and unhygienic conditions during delivery (especially in resource-poor settings). On the contrary, other studies have reported a higher occurrence of LOS than EOS [22, 23]. The bacteriological profile of EOS differs from that of LOS as the mode of infection is different [24]. Early-onset neonatal sepsis can occur by ascending infection from the mother's cervix or passage of the baby through a colonized birth canal [15].

A high prevalence of Gram-positive microorganisms (72.0%) was found compared to Gram-negative (28.0%) organisms. This finding is similar to a related study conducted in a Ghanaian tertiary hospital, where Gram-positive microorganisms had preponderance over Gram-negative organisms [10]. A study conducted at a neonatal intensive care unit in China found that Gram-positive organisms were responsible for a greater proportion of EOS (83.3%) [25]. Studies have reported a high prevalence of coagulase-negative staphylococci among neonatal blood cultures [26, 27]. Reports from these studies corroborate our finding in this study, as coagulase-negative staphylococci (*S. epidermidis* and *S. haemolyticus*) were the predominant isolated bacteria. Coagulase-negative staphylococci have been found to play a significant role in EOS in neonates, especially those with low birth weight and gestational age [28]. High percentage of coagulase-negative staphylococci identified in blood cultures can also be attributed to contamination from skin flora of neonates due to poor skin disinfection techniques. Thus, the high rate of isolation of coagulase-negative staphylococci from our study may be due to contamination.

In this present study, *Staphylococcus epidermidis*, *Staphylococcus aureus,* and *Klebsiella pneumoniae* were the common etiological agents of LOS. *Klebsiella spp*. were the predominant Gram-negative species isolated in this study, and this corroborates previous reports [29, 30]. Reports from a study in Nepal indicated that *Enterobacter spp*. (15.0%), *Acinetobacter spp*. (12.0%), and *Escherichia coli* (12.0%) were the commonest isolated Gram-negative organisms in LOS [31]. Furthermore, a study conducted in South Africa identified *Acinetobacter baumannii, Klebsiella pneumoniae,* and *Escherichia coli* as the predominant Gram-negative bacteria, together with a few *Pseudomonas aeruginosa* and *Enterobacter spp.* [26]. Considering that the pathogens most often implicated in neonatal sepsis differ between geographic regions, countries, and facilities, the findings in this study are relevant.

In this study, Gram-positive isolates exhibited high sensitivity to amikacin and “reserved drugs” such as meropenem, piperacillin/tazobactam, and vancomycin. Also, coagulase-negative staphylococci showed high sensitivity to meropenem, vancomycin, piperacillin/tazobactam, and amikacin. Almost all of the Gram-positive isolates were susceptible to vancomycin. Drugs such as meropenem and piperacillin/tazobactam should not be used indiscriminately as bacteria's resistance to these drugs may increase morbidity and mortality in neonates [32]. The present study showed high resistance among Gram-positive isolates to cotrimoxazole (89.2%), ampicillin (85.0%), and penicillin (84.9%). The resistance of Gram-positive isolates ranged from 45.0% to 89.2% for flucloxacillin, amoxiclav, ampicillin, penicillin, and cotrimoxazole. Further analysis of the antibiotic susceptibility data showed that coagulase-negative staphylococci exhibited resistance to ampicillin (85.0%), penicillin (89.2%), and cotrimoxazole (90.1%). This confirms reports from a related study among neonates where *Staphylococcus epidermidis* and *Staphylococcus haemolyticus* showed poor sensitivity (13.5%) to ampicillin [33]. High resistance exhibited by Gram-positive organisms to ampicillin and penicillin corroborates other similar studies [34–39]. Further analysis showed that *Klebsiella* isolates were resistant to cefotaxime, a commonly used second-line antibiotic. Lubell et al. [40] reviewed the literature regarding antibiotic susceptibility patterns of community-acquired pathogens causing neonatal sepsis in sub-Saharan Africa and Asia. The two common pathogens, *S. aureus* and *Klebsiella spp*., exhibited high rates of resistance to almost all commonly used antibiotics (ampicillin, ceftriaxone, chloramphenicol, cotrimoxazole, macrolides, and gentamicin).

As there is some level of antibiotic resistance observed in the current study, it is necessary for routine microbial analysis of samples and their antibiogram. Although the current study showed that bacteria were resistant to some penicillin types at the Greater Accra Regional Hospital, the 20^th^ edition of the WHO Model List of Essential Medicines [41] also ought to be considered in order to address the burden of antimicrobial resistance. This list has ACCESS (affordable and safe antibiotics that should be widely available), WATCH (antibiotics with higher resistance potential recommended as first choice only for a few specific indications or as second choice), and RESERVE (antibiotics that should be restricted for use in specific patients when all other alternatives have failed).

Findings from this study indicate that continuous surveillance of causative agents of neonatal bloodstream infections is required to guide facility and national treatment guidelines. In addition, studies of this nature provide guidance for the development of local antibiograms, which can aid in the empiric and rational treatment of neonatal sepsis. Education on adherence to treatment guidelines and periodic review of antibiotic policies are relevant in the quest to reduce antimicrobial resistance.

## Figures and Tables

**Table 1:** Bacteria isolated from blood cultures of neonates with sepsis (*n* = 528) at the Greater Accra Regional Hospital.

Isolate	Early-onset sepsis	Late-onset sepsis	Total (%)
	Frequency	Frequency	
Gram-positive			
*Staphylococcus epidermidis*	275	42	(317) 60.0%
*Staphylococcus haemolyticus*	26	11	(37) 7.0%
*Staphylococcus aureus*	5	16	(21) 4.0%
*Enterococcus spp.*	5	0	(5) 0.9%

Gram-negative			
*Klebsiella spp.*	33	40	(73) 13.6%
*Escherichia coli*	4	17	(21) 4.0%
*Enterobacter spp.*	2	13	(15) 2.9%
*Pseudomonas spp.*	5	23	(28) 5.4%
*Serratia spp.*	1	3	(4) 0.8%
*Pasteurella spp.*	1	3	(4) 0.8%
*Acinetobacter*	0	3	(3) 0.6%

Total	**357**	**171**	**(528) 100%**

## Data Availability

The data used to support the findings of this study are available from the corresponding author upon request.
